# Clinical and metabolic response to soy administration in older women with metabolic syndrome: a randomized controlled trial

**DOI:** 10.1186/s13098-019-0441-y

**Published:** 2019-06-20

**Authors:** Afsaneh Bakhtiari, Karimollah Hajian-Tilaki, Shabnam Omidvar, Fatemeh Nasiri-Amiri

**Affiliations:** 1 Mobility Impairment Research Center, Health Research Institute, Babol University of Medical Sciences, Babol, Iran; 2 Department of Biostatistics and Epidemiology, Faculty of Medicine, Babol University of Medical Sciences, Babol, Iran; 3 Social Determinants of Health Research Center, Health Research Institute, Babol University of Medical Sciences, Babol, Iran; 4 Infertility and Health Reproductive Research Center, Health Research Institute, Babol University of Medical Sciences, Babol, Iran; 5 Department of Midwifery, Faculty of Nursing and Midwifery, Babol University of Medical Sciences, Babol, Iran

**Keywords:** Metabolic syndrome, Elderly women, Soy protein, Soy-nut

## Abstract

**Background:**

There are many studies on the health effects of soy, only a few describe the effects of the simultaneous use of two types of soy on multiple components of metabolic syndrome (MetS). The present study was designed to determine the effects of roasted soy-nut and textured soy protein (TSP) intake on clinical and metabolic status of older women with MetS borderline parameters.

**Method:**

This randomized, single-blind, controlled clinical trial included 75 women ≥ 60 years old with a diagnosis of MetS based on ATP III criteria. The participants were randomly allocated into three groups of 25 people; soy-nut, TSP and control groups for 12 week. Fasting blood samples were taken at the beginning and end of the trial to compare the metabolic responses. All participants provided three dietary records and physical activity records during the intervention. We used the Kolmogorov–Smirnov, ANOVA, ANCOVA, paired-t test, and the Generalized Linear Model (GLM) repeated measures analysis.

**Results:**

Dietary intake and physical activity of the participants in two groups were not significantly different. After 12 weeks of intervention the participants who received soy-nut had a significant decrease in total cholesterol (TC) (p < 0.001), low density lipoprotein, very low density lipoprotein, apolipoprotein B100, fasting blood glucose, insulin (p < 0.05), HOMA-IR, malondialdehyde (MDA) (p < 0.01) level. Morever, a significant increase in total antioxidant capacity (TAC) (p < 0.01) level compared with the control group. At the same time, the TSP brought significant decrease only in TC, insulin, MDA (p < 0.05) level and a significant increase in total TAC (p < 0.05) level. We did not find any significant effect in intervention groups, on apolipoprotein AI, triglyceride (TG), high density lipoprotein (HDL-C), TG/HDL, C-reactive protein and fibrinogen levels after intervention.

**Conclusion:**

Short-term intakes of roasted soy-nut and TSP have shown to improve the lipid profiles, markers of glucose intolerance and oxidative stress; although the roasted soy-nut was more effective than TSP. Therefore, a moderate daily intake of roasted soy-nut as snacks or TSP as a meal complement by individuals with borderline parameters of MetS can be a safe and a practical modality to avoid the progression of the disease as well as to limit the side effects of drug intake.

*Trial registration* MUBABOL.REC.1388.1

**Electronic supplementary material:**

The online version of this article (10.1186/s13098-019-0441-y) contains supplementary material, which is available to authorized users.

## Introduction

Metabolic syndrome (MetS), and its various features involving all body organs in the pathological process, is a major public health problem that causes the global burden of disease. The main concern of MetS is that each of its individual components is associated with increased cardiovascular disease (CVD) risks, while the presence of MetS greatly augments CVD morbidity and mortality [1]. Mets is caused by increase of visceral fats which promotes synthesis of proinflammatory cytokines, which in turn results in the development of reactive oxygen species (ROS), thrombotic and atherogenic factors, which are all conditions with Mets [2]. Meanwhile, existing data suggest that the incidence of MetS is rising at an alarming rate in the world [3] and Iran [4]. The prevalence of MetS has been reported to be higher in Iran in compare to other Asian and European countries [1, 3, 4]. The prevalence of the MetS increases with age, and reaches the peak in the fifth decade of life. According to the definition of ATPIII, more than half of the population aged 60 years and higher, have MetS in Iran; prevalence in elderly women is higher than men [4].

Menopausal status and aging are linked to some components of MetS. With increase in age, decrease in muscle mass occurs, which this leads to decreased peripheral glucose uptake and increased oxidative stress. This also causes exacerbation of impaired insulin secretion and progression of atherosclerosis and ultimately hypertension [5]. Thus, treatment of MetS can prevent from disability and will promote healthy aging.

However, treatment of MetS must address the multipathological process of MetS, with each component identified and aggressively targeted for treatment. In theory, it is possible to treat each of the symptoms of MetS using the present optimal method or pharmacological agent, as this will result in treating obesity, hypertension, dyslipidaemia, hyperglycaemia, and exaggerated platelet aggregation, each with a different mode. This may lead to separate treatments for numerous disorders [2]. Nevertheless, the focus of today’s efforts is to discover and apply methods that simultaneously mitigate several morbid features of MetS, by treating the underlying link or cause rather than by treating each discrete manifestation [6]. There is evidence which indicate that modifications of dietary habits and lifestyle patterns are the first-line therapy to prevent and treat MetS. It is known that soy can have an important role in preventing vascular diseases through its effects on insulin resistance, inflammatory process affecting vascular disease, and oxidative stress. Soy consumption has been reported to beneficially affect features of MetS in animal models [7–10], and also in humans to some extent [11, 12]. Several studies have consistently reported the effects of soy on cardiovascular risks such as, lipid profile [13, 14], glycemia status [15, 16], proinflammatory [17, 18], prothrombotic [19, 20] and oxidative stress markers [21, 22]; however, not all studies agree on these effects [23–27].

Soybeans are the most excellent source of unsaturated fatty acids, dietary fiber, isoflavones, and antioxidants. Soybeans are also fiber-rich, nutrient-dense, and are high-quality sources of protein; all requirements of a dietary intervention to reduce cardiometabolic risk. The content and bioavailability of protein, fats, minerals and isoflavones in dry beans and soy foods vary according to their processing methods and phytate content.

Soy nuts are made by soaking dry soybeans and baking them in one layer on a well-oiled cookie sheet at around 350 °F (190 °C) during 30 to 50 min until well browned. Soy proteins are also divided into different classes according to their production methods. Textured Soy Protein (TSP) is manufactured by forming doughs from high nitrogen solubility index, dehulled and defatted soy flour with water in a screw-type extruder and heated with steam. Therefore TSP is high in protein and low in fat [28]. Texturization of the flour, leads to a decrease of the isoflavone content in the TSP. Dehulling was also shown to be a way of decreasing the isoflavone content of the flour prepared from raw seeds. Heat treatment of soy increases the digestibility of the protein and enhances the nutritional quality of soybeans. The soy cotyledon storage proteins can be extracted most efficiently from dehulled and defatted soybeans that have undergone only a minimal heat treatment so that the protein is close to being native or undenatured. All of these processes result in a product that is 70% protein, 20% carbohydrates (2.7 to 5% crude fiber), 6% ash and about 1% oil; but the solubility may differ [29].

Considering the health effects of soybeans, as well as the impact of the production process on some of the soybean’s active ingredients, the question arises as to how the health effects of soy-processed products compare to other products. For instance, roasted soy-nuts are made from whole soy beans which are non-processed products. In contrast, TSP comes in the form of flakes, like of ground meat that comes from manufacturing process. Therefore, this study was specifically designed to determine the effects of roasted soy-nuts in the natural state of soybean against TSP, as a processed soy product on the full profile of MetS in women aged 60 years and higher. The results of this study can be taken as a practical way to reduce the need for medical treatment in elderly women with MetS through simultaneous improvement of multiple metabolic disorders.

## Method and material

### Participants

Seventy-five peoples with MetS whose age ranged between 60 and 70 years were recruited for this randomized, single-blind, parallel controlled trial. To determine the sample size, we applied a randomized clinical trial sample size formula considering type I (α) and type II errors (β) of 0.05 and 0.20 (power 80%), respectively; in order to detect the effect size of 0.8 in metabolic response between comparison groups. Thus allocated sample size was 21 subjects for each group and by considering a %15 dropout rate, 75 participants were included in the study.

### Recruitment and screening

The volunteers were recruited by advertisements in the Rural Health Centers affiliated with the Babol University of Medical Sciences (BUMS), Iran. Thus, one cohort of 306 volunteers was initially screened via personal basic interview using a prescreening evaluation, including their medical history, medication, lipid and glucose records, abdominal obesity and hypertension. A total of 142 volunteers were excluded and 164 subjects who meet the initial criteria was be invited to determine their eligibility by assessment of central obesity, blood pressure, fasting blood glocuse (FBG), triglyceride (TG) and high density lipoprotein (HDL-C). Among them, 75 eligible subjects with a diagnosis of MetS based on ATPIII criteria [4], and without receiving any medications for the treatment of diabetes, hypertension, and hyperlipidemia were included in the study. However individuals who received medication because of the following lab results LDL-C above 160 or 189 mg/dl depending on the number of CVD risk factors, TG ˃ 500 mg/dl, FBG ≥ 126 mg/dl, and blood pressure ≥ 140/90 mm Hg didn’t enter the study [30–32].

Exclusion criteria were a history of kidney, liver, thyroid, infectious diseases, cancer, and CVD, and taking antibiotic, dietary supplements or probiotic supplements during the previous 3 mo. All procedures were carried out on the basis of ethics standards of the responsible committee on human experimentation (institutional and national) and to the Declaration of Helsinki. In addition, the ethics committee of BUMS approved the study (NO.:MUBABOL.REC.1388.1). All participants provided an informed written and signed consent form. All patients provided written informed consent.

### Study design

In the present study, the participants were randomly allocated into three groups to receive soy-nut (n = 25) or TSP (n = 25) or control group (n = 25) using a table of random numbers generated by Microsoft Excel. Randomization and allocation was concealed from those who did blood biochemical and statistical analysis. Soy used in this study, comprised of roasted soy-nut and TSP, both provided by the Max Soy Company in Tehran, Iran. The participants were provided with 2 weekly supply of soy in 490 gr bags. A daily dosage of 35 g of roasted soy-nut or TSP was consumed by the intervention groups during the 12-week intervention. The control group received no intervention, since the participants in the intervention groups were consuming natural soy products not pills, so it was impossible to apply any placebo for the control group.

The amount of soy used in this study was selected based on previous studies have shown that soy isoflavones amounts equal to or greater than 90 mg per day can reduce insulin levels in healthy postmenopausal women [33, 34]. This is consistent with the amount of isoflavone used in this study (96.2 mg in TSP and 117.2 mg in roasted soy-nut groups). The nutrient composition of soy consumed in the study, based on Max Soy Company analysis, is shown in Table 1.

**Table 1:** Nutrient composition of soy used in the study

Nutrients/35 g	Soy-nut	TSP
Energy (Kcal)	176	122
Protein (g)	13.8	18.2
Fat (g)	8.7	0.45
Total carbohydrate (g)	11.5	11.4
Fiber (g)	11.9	10.5
Sodium (mg)	11.9	10.5
Calcium (mg)	54.6	46.5
Magnesium (mg)	50.7	47.1
Potassium (mg)	514.5	427.5
Isoflavones (mg)	117.2	96.2
Diadzein (mg)	47.6	38.5
Genistein (mg)	60.2	48.8
Glycitein (mg)	9.45	8.9

TSP: Textured soy protein

The participants in the TSP group were instructed on the preparation of their TSP meal. First, they have to soak the TSP with warm water for about 2–3 min and drained it. Next, they have to cook the TSP with turmeric powder and lime juice for 2 min. The prepared TSP meal was to be taken in a portion at breakfast or around mid-morning (10:00 am to 10:30 am). Lastly, they were strongly advised to completely take all the prepared TSP to avoid any possible errors in the results. Along with soy-nut and TSP bags, the participants were also given graduated beakers of 100 ml and 200 ml for estimating the quantity of soy-nut and TSP to be consumed, retrospectively.

**Fig. 1:**
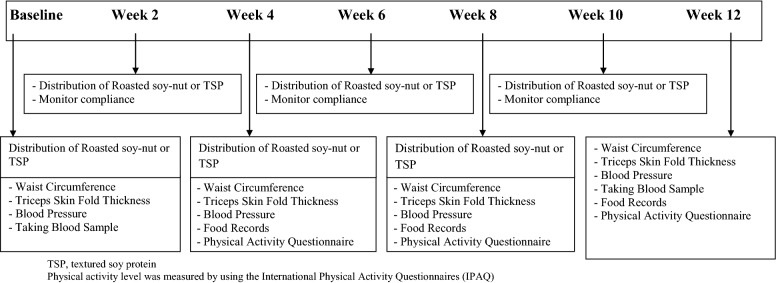
Data collection schedule during 12-week intervention period

The daily diet and physical activity habits of the participants remained unchanged as the research was directed towards the effects of soy on metabolic indices of the human body. Compliance to soy consumption was monitored by asking participants to return the soy bags. The returned bags were weighted to measure the remaining soy, if any. Adherence was also monitored by regular attendance at the bi-weekly meetings and by monthly analysis of 3-day food records and physical activity questionnaire. All participants provided three physical activity records (for the last 7 days of each month) and three dietary records (for three consecutive days on study weeks) to ensure that they maintained their usual diet and physical activity during the intervention. Both dietary and physical activity records were taken at weeks 4, 8 and 12 of the intervention (Fig. 1). Food intake analyzed by Nutritionist IV software (First Databank, San Bruno, CA).

### Outcomes

In this study, the primary outcome was the changes from the baseline in lipid profile [total cholesterol (TC), low density lipoprotein (LDL-C), very low density lipoprotein (VLDL-C),HDL-C, TG, apolipoprotein B100 (Apo B100) and apolipoprotein AI (APO AI), glycemia status [FBG, serum insulin, homeostasis model of assessment insulin resistance (HOMA-IR), TG/HDL], oxidative stress [malondialdehyde (MDA), total antioxidant capacity (TAC)], C-reactive protein (CRP), fibrinogen, also waist circumference (WC), triceps skin fold (TSF), systolic blood pressure (SBP) and diastolic blood pressure (DBP). Fasting blood samples were obtained at the baseline and study endpoint at the Biochemical Research Laboratory of the BUMS in an early morning after an overnight 12-h fast.

Three fully- skilled specialists did all blood sample analyses in the biochemical research laboratory of the BUMS. Each specialist was responsible for a specific blood profile analysis; lipid and glucose analysis, Elisa test (Apo AI, Apo B100, CRP, insulin), and TAC and MDA tests. Ten ml of venous blood was drawn; 1.8 ml of the blood samples was collected in citrated tubes for measuring fibrinogen. The rest of the blood samples were collected into test tubes. Blood samples were left at room temperature until they clotted. Clots were gently separated from the tubes by use of a wooden applicator and then the blood samples were centrifuged for 10 min at 2000 rpm to obtain the serum samples, within 15 min of collection. Serum samples were separated into microtubes and then they were frozen at − 80 °C until further analysis.

Fasting serum TC and TG concentrations were measured using Elitech kit from France, LDL-C, HDL-C, VLDL-C and FBG by Pars Azmoon kit from Iran. The lipid profiles and FBG were assayed on Mindray—BS 300, chemistry Autoanalyzer (Mindray—BS 300, Nanshan, Shenzhen, China). TG/HDL-C Ratio was calculated and used as an indicator of insulin resistance. To determine serum apoB100, apoAI, insulin and hs-CRP concentrations, we used ELISA kits (Diagnostic Mabteck AB, Sweden, DiaPlus Canada Inc, Montreal, Canada and AccuBind ELISA Kit, Monobind Inc., Costa Mesa, USA, respectively). The homeostasis model of assessment insulin resistance (HOMA-IR) was determined based on suggested formulas [35]. Fibrinogen was measured 1 h after sampling using MAHSA-YARAN kit from Iran, through quantitative determination of plasma fibrinogen based on Clauss method (clotting method) (WHO, 1985). MDA as direct marker of systemic oxidative stress and peroxidation lipid was evaluated by TBARS method (thiobarbituric acid reactive substances) according to the method of Ruiz-Ramos et al. [36]. Plasma TAC was quantified by using the FRAP method modified by Benzie and Strain [37].

For measuring the weight, a SECA Digital Scale (SECA, British Indicators Ltd., United Kingdom) was applied, which was calibrated with 1 kg of known weight and with the accuracy of 100 g. For weight, participants were requested to wear normal light clothes. Standing position of participants during weight and height measurement was straight. Participants were requested to take off their shoes for weight and height measures to be accurate [38]. For height measurement, participants stood upright on the board of the stadiometer while their back positioned to the vertical background of the stadiometer [38]. BMI was calculated as weight (in kg) divided by height in meters squared [38].

WC was measured as the width between the bottom rib and the top of the hip of the participants using a metric measuring tape in standing position. TSF was measured by a skinfold caliper (Pooyaye Armaghan Co., Iran) at the upper arm mid-point mark on the posterior surface of the right upper arm between the acromion process of scapula and the olecranon process. Middle point was marked with the elbow flexed at 90° and measurement of the thickness of a fold of skin and the underlying subcutaneous tissue was made with the arm hanging loosely at the side of the body with the skinfold caliper Blood pressure was analyzed using the ALPK2 Aneroid model, Sphygmomanometer, (Tanaka Sangyo, Co. LTD. Tokyo, Japan). The secondary outcome included soy intervention complications (most complaints include soy allergies, mild constipation, nausea, bloating, burning and stomach ache), participants’ compliance, and soy tolerance and comparison in the two groups.

### Statistical analysis

Normality of variables of interest was tested by Kolmogorov–Smirnov. To detect differences in general characteristics, dietary intakes and physical activity in the groups, we used X^2^ and ANOVA test. To determine the effects of soy administration on markers of lipid profile, apoB100 and AI, glucose intolerance, oxidative stress, CRP, fibrinogen, also WC, TSF and blood pressure, we used one-way repeated measures analysis of variance. Within-group comparisons (endpoint vs. baseline) were done based on the paired-samples t test. Analysis of Covariance (ANCOVA) was also run to determine the effect of the intervention on the treatment groups after removing the variances for pre-treatment levels of the variables. The Generalized Linear Model (GLM) repeated measures analysis, two factor mixed design was applied to detect the changes in mean of the WC, TSF, SBP and DBP of the participants during the 12-week intervention. Statistical analysis was performed using SPSS version 17 software (SPSS Inc., Chicago, IL). The two-tailed p value less than 0.05 were considered significant.

## Results

All participants completed the entire study. We found that, the rate of compliance in the present study was high, as more than 90% of soy bags were taken throughout the study in both groups intervention. Both the soy-nut and TSP were well tolerated. There was no serious complaint on the consumption of soy except for the cause of flatulence in five of the participants in the TSP group.

Baseline characteristics of the participants are presented in Table 2. The participants did not have significant differences with regard to any baseline variables, as well as in terms of dietary intakes and physical activity throughout the intervention. Results of the food record analysis are presented in Additional file 1: Table S1. After having taken out the energy and fiber from the soy supplementations, there were no significant differences in energy, protein, carbohydrate, saturated fat, monounsaturated fat, polyunsaturated fat and dietary fiber among the three groups at baseline and after intervention. As well as mean of dietary intake of the participants during the 12-week intervention was reported in Additional file 1: Table S2. Additional file 1: Table S3 shows different domains of physical activity of the participants according to international physical activity questionnaire (IPAQ) at baseline and after intervention. There was no significant difference in physical activity level at the baseline and after intervention in the groups.

**Table 2:** Baseline participants’ characteristics^a^

Variables	Soy-nut (n = 75)	TSP (n = 75)	Control (n = 75)
Age (years)	63.8 ± 2.82	64.6 ± 2.91	64.1 ± 2.81
Age of menopause (years)	48.2 ± 3.91	47.7 ± 4.72	48.6 ± 3.62
Currently in menopause (years)	15.5 ± 3.64	16.8 ± 61	15.5 ± 3.75
SBP (mm Hg)	127.3 ± 4.41	127.6 ± 4.48	127.4 ± 4.64
DBP (mm Hg)	79.4 ± 6.47	80.6 ± 4.34	81.4 ± 6.15
Live with			
Alone	4 (16)	2 (8)	2 (8)
Husband	19 (76)	19 (76)	20 (80)
Children	2 (8)	4 (16)	3 (12)
Economic status			
Dependent	2 (8)	1 (4)	2 (8)
Independent	23 (92)	24 (96)	23 (92)

TSP: Textured soy protein; SBP: systolic blood pressure; DBP: diastolic blood pressure; BMI: body mass index

Obtained from ANOVA and χ^2^ test

^a^Values are mean ± SD or numbers of participants (percentages)

After 12 weeks of intervention, the participants who received soy-nut had significantly decreased TC (p < 0.001), LDL-C (p < 0.05), VLDL-C (p < 0.05), apoB100 (p < 0.05), FBG (p < 0.05), insulin (p < 0.05), HOMA-IR (p < 0.01), MDA (p < 0.01) levels and a significantly increased in TAC (p < 0.01) level compared with the control group. At the same time, the TSP decreased significantly TC (p < 0.05), insulin (p < 0.05), MDA (p < 0.05) levels and a significant increase in TAC (p < 0.05) level. We did not find significant effects of soy consumption, on apo AI, TG, HDL-C, TG/HDL, CRP and fibrinogen levels after intervention (Table 3). Mean changes within groups showed significant inter-individual changes for all variables of lipid profiles; glycemia status (p < 0.001) and oxidative stress (p < 0.001) markers in both intervention groups (p < 0.001). The soy-nut group experienced more changes, though the changes were not significant between intervention groups on the blood markers except for glycemiamarkers (Table 4). After adjusting for blood biomarkers baseline values by Analysis of Covariance, the results remained constant.

**Tables 3:** Metabolic status at the baseline and end of trial

Metabolic indicators	Baseline				End of trial			
	Soy-nut	TSP	Control	P value	Soy-nut	TSP	Control	P value
TC (mg/dl)	229.9 ± 5.13	229.4 ± 5.84	233.2 ± 5.20	0.87	200.7 ± 4.82^a^	205.2 ± 5.55^a^	224.5 ± 5.21	< 0.001
TG (mg/dl)	212.1 ± 8.23	211.9 ± 8.86	212.6 ± 10.65	0.99	199.8 ± 8.65	200.3 ± 9.09	208.5 ± 10.79	0.77
HDL-C (mg/dl)	44.2 ± 1.38	43.0 ± 0.98	44.2 ± 1.58	0.78	46.3 ± 1.17	44.8 ± 0.93	43.7 ± 1.71	0.35
LDL-C (mg/dl)	154.0 ± 3.28	154.7 ± 5.80	152.3 ± 6.01	0.95	131.0 ± 5.42^a^	134.5 ± 5.65	151.5 ± 6.51	< 0.05
VLDL-C (mg/dl)	41.7 ± 1.56	42.4 ± 1.77	42.4 ± 2.19	0.95	34.5 ± 1.83^a^	37.4 ± 2.03	42.0 ± 2.28	< 0.05
Apo AI (g/l)	1.8 ± 0.20	1.7 ± 0.22	1.6 ± 0.22	0.77	2.0 ± 0.19	1.9 ± 0.22	1.6 ± 0.21	0.31
Apo B_100_(g/l)	1.5 ± 0.09	1.5 ± 0.10	1.5 ± 0.17	0.96	1.2 ± 0.07^a^	1.2 ± 0.09	1.6 ± 0.16	< 0.05
FBG (mg/dl)	104.8 ± 2.01	104.3 ± 2.32	102.5 ± 2.49	0.75	90.4 ± 2.18^a^	97.8 ± 2.34	99.6 ± 2.83	< 0.05
Insulin (µIU/ml)	12.8 ± 0.79	11.6 ± 0.95	13.4 ± 0.9	0.36	10.8 ± 0.79^a^	10.8 ± 0.85^a^	13.8 ± 0.90	< 0.05
HOMA-IR	3.4 ± 0.25	3.0 ± 0.27	3.4 ± 0.25	0.51	2.5 ± 0.21^a^	2.6 ± 0.23	3.4 ± 0.24	< 0.01
TG/HDL	4.9 ± 0.27	5 ± 0.25	4.9 ± 0.34	0.99	4.41 ± 0.24	4.5 ± 0.24	5 ± 0.39	0.35
CRP (µg/ml)	3.2 ± 0.37	3.1 ± 0.45	3.0 ± 0.49	0.97	2.9 ± 0.38	3.0 ± 0.39	3.0 ± 0.33	0.98
Fibrinogen (mg/dl)	316.7 ± 6.89	316.5 ± 8.88	314.2 ± 9.37	0.97	297.8 ± 6.07	302.0 ± 6.37	306.4 ± 6.59	0.63
MDA (µmol/l)	4.9 ± 0.36	4.9 ± 0.17	5.3 ± 0.28	0.48	4.2 ± 0.35^a^	4.3 ± 0.19^a^	5.3 ± 0.25	< 0.001
TAC (µmol/l)	1302.0 ± 45.36	1305.3 ± 45.37	1305.1 ± 40.09	0.99	1516.3 ± 39.70^a^	1503.4 ± 35.28^a^	1350.2 ± 47.38	< 0.01

TSP: Textured soy protein; SBP: systolic blood pressure; DBP: diastolic blood pressure; TC: total cholesterol; TG: triglyceride; HDL-C: high density lipoprotein; LDL-C: low density lipoprotein; VLDL-C: very low density lipoprotein; Apo AI: apolipoprotein AI; Apo B100: apolipoprotein B100; FBG: fasting blood glucose; HOMA-IR: homeostasis model of assessment insulin resistance; TG/HDL-C: triglyceride/high density lipoprotein; MDA: malondialdehyde; TAC: total antioxidant capacity; CRP: C-reactive protein

Values are mean ± standard error

^a^There was a significant difference in the treatment groups compared with control group (post hoc ANOVA)

**Tables 4:** Mean changes in metabolic indicators at the baseline and end of trial

Metabolic indicators	Mean change			
	Soy-nut	TSP	Control	P value
TC (mg/dl)	− 29.2 ± 3.56^a,c^	− 24.3 ± 3.54^a,c^	− 8.7 ± 5.47	< 0.01
TG (mg/dl)	− 12.2 ± 1.86^c^	− 11.6 ± 1.95^c^	− 4.1 ± 1.12	0.14
HDL-C (mg/dl)	2.1 ± 0.49^c^	1.7 ± 0.64^c^	− 0.57 ± 1.46	0.10
LDL-C (mg/dl)	− 23.1 ± 2.03^a,c^	− 20.1 ± 3.00^a,c^	− 0.80 ± 3.84	< 0.001
VLDL-C (mg/dl)	− 7.1 ± 1.11^a,c^	− 5.0 ± 0.67^a,c^	− 0.37 ± 1.23	< 0.001
Apo AI (g/l)	0.19 ± 0.03^a,c^	0.18 ± 0.03^a,c^	− 0.02. ± 0.01	< 0.01
Apo B_100_(g/l)	− 0.31 ± 0.04^a,c^	− 0.28 ± 0.01^a,c^	0.06 ± 0.05	< 0.001
FBG (mg/dl)	− 14.4 ± 1.10^b,c^	− 6.5 ± 0.60^c^	− 2.8 ± 1.54	< 0.001
Insulin (µIU/ml)	− 2.0 ± 0.29^b,c^	− 0.8 ± 0.20^a,c^	0.4 ± 0.22	< 0.001
HOMA-IR	− 0.9 ± 0.08^b,c^	− 0.4 ± 0.06^a,c^	− 0.02 ± 0.07	< 0.001
TG/HDL	− 0.5 ± 0.08^a,c^	− 0.4 ± 0.09^a,c^	0.03 ± 0.21	< 0.01
CRP (µg/ml)	− 0.25 ± 0.16	− 0.16 ± 0.17	− 0.04 ± 0.32	0.8
Fibrinogen (mg/dl)	− 18.8 ± 8.98	− 14.5 ± 9.53	− 7.8 ± 6.24	0.65
MDA (µmol/l)	− 0.75 ± 0.04^a,c^	− 0.63 ± 0.07^a,c^	− 0.01 ± 0.08	< 0.001
TAC (µmol/l)	214.2 ± 20.02^a,c^	198.0 ± 23.47^a,c^	45.1 ± 19.34	< 0.001

Values are mean ± standard error

TSP: Textured soy protein; SBP: systolic blood pressure; DBP: diastolic blood pressure; TC: total cholesterol; TG: triglyceride; HDL-C: high density lipoprotein; LDL-C: low density lipoprotein; VLDL-C: very low density lipoprotein; Apo AI: apolipoprotein AI; Apo B100: apolipoprotein B100; FBG: fasting blood glucose; HOMA-IR: homeostasis model of assessment insulin resistance; TG/HDL-C: triglyceride/high density lipoprotein; MDA: malondialdehyde; TAC: total antioxidant capacity; CRP: C-reactive protein

^a^There was a significant difference in the treatment groups compared with control group (post hoc ANOVA)

^b^There was a significant difference in the soy-nut group compared with TSP and control groups (post hoc ANOVA)

^c^For significant changes after-before (paired t-test)

**Fig. 2:**
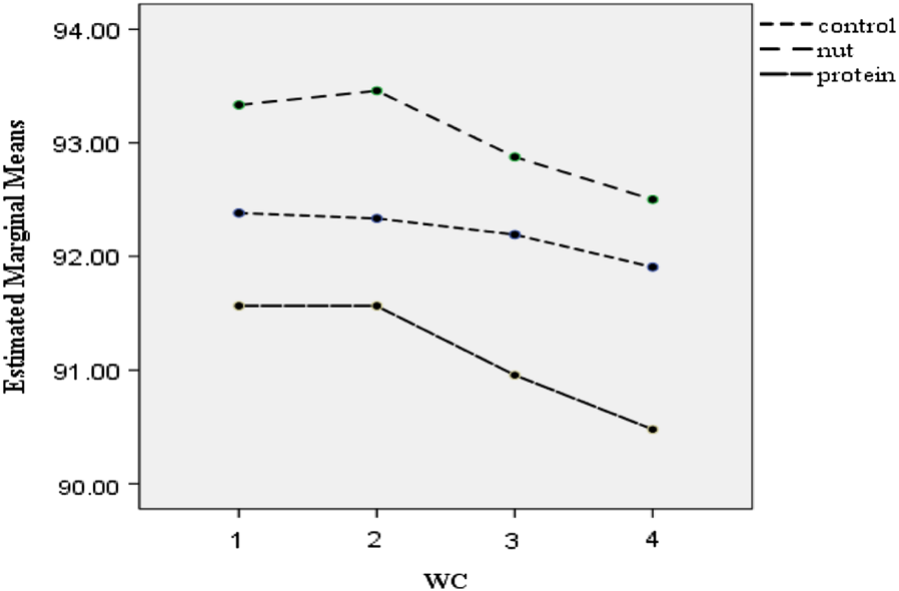
Waist circumference mean for intervention and control groups by time interaction

**Fig. 3:**
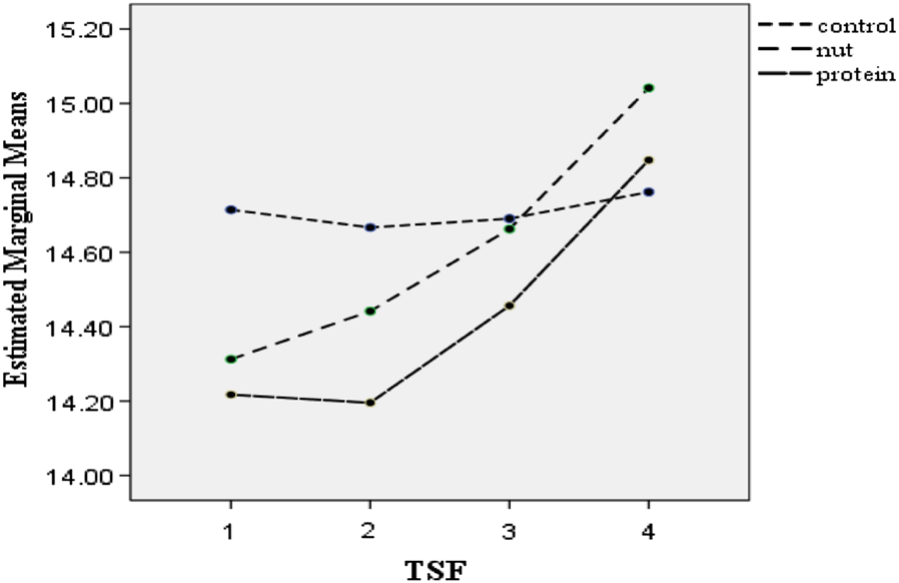
Triceps skin fold mean for intervention and control groups by time interaction

**Fig. 4:**
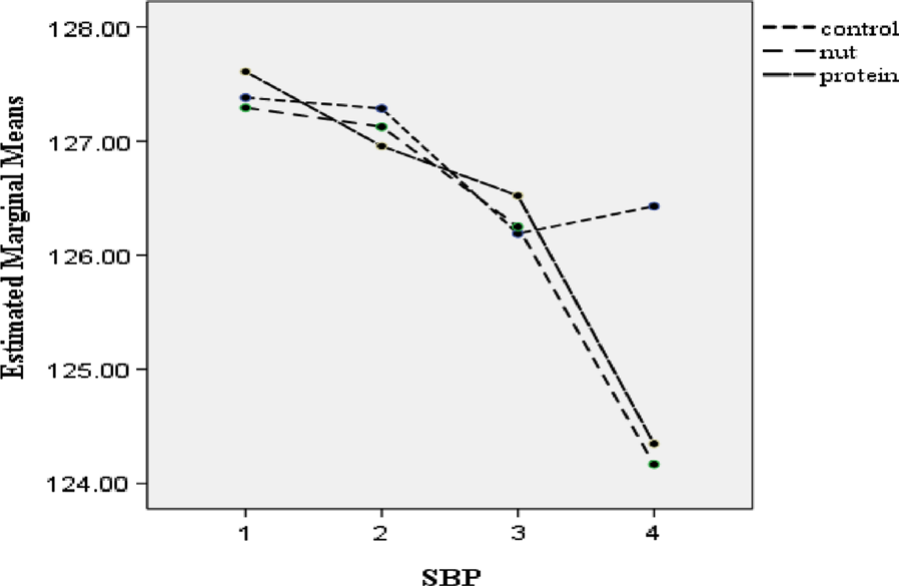
Systolic blood pressure mean for intervention and control groups by time interaction

**Fig. 5:**
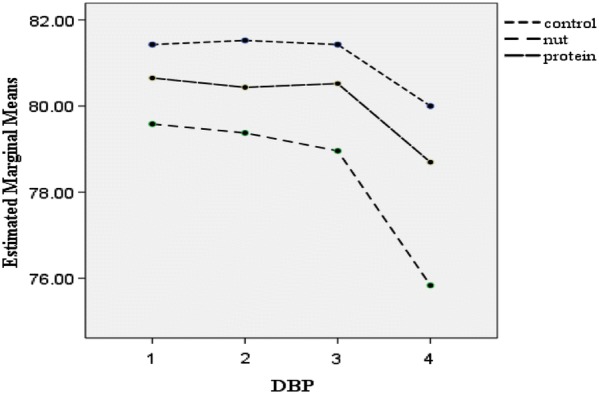
Diastolic blood pressure mean for intervention and control groups by time interaction

**Fig. 6:**
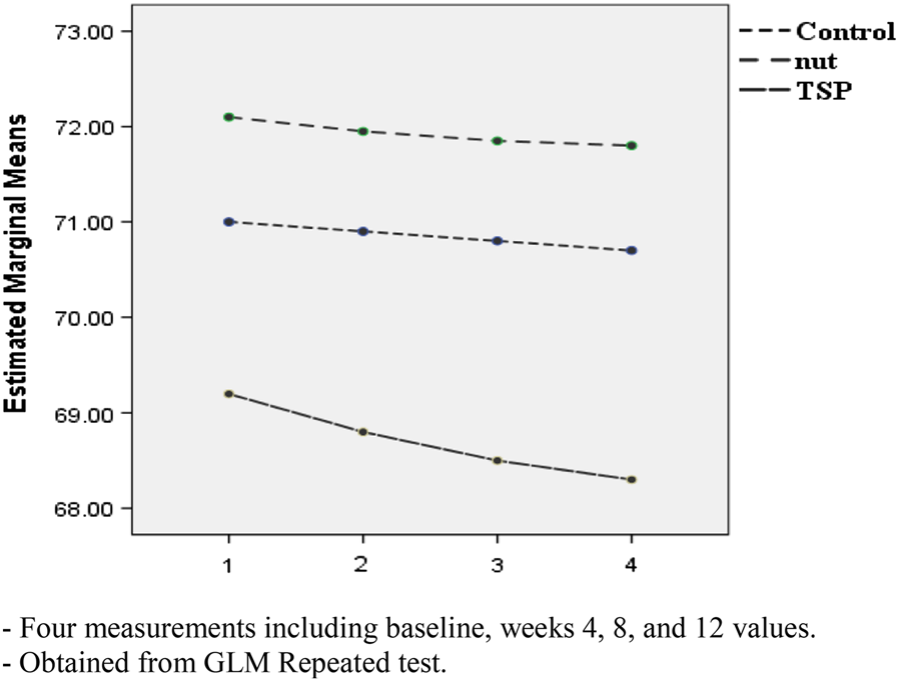
Weight mean for intervention and control groups by time interaction

**Fig. 7:**
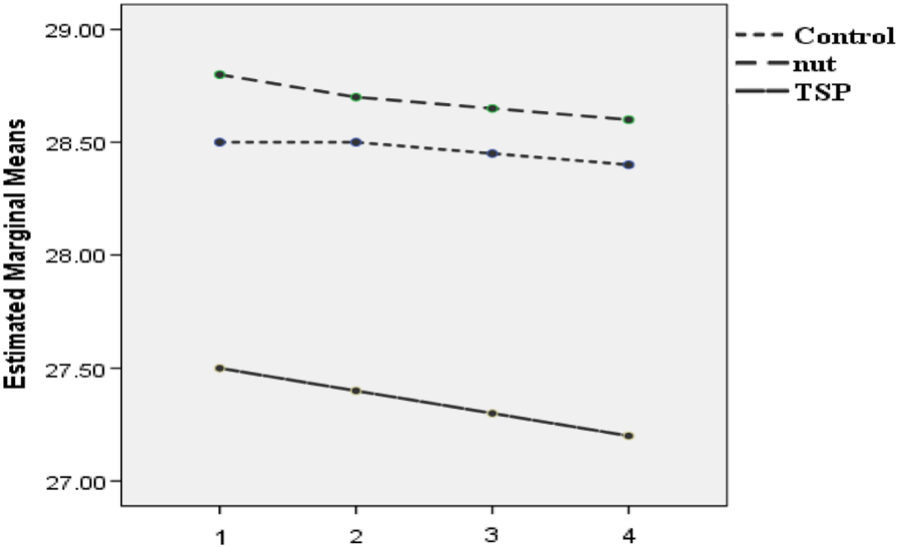
BMI mean for intervention and control groups by time interaction

Post-hoc GLM repeated measures, did not show any significant effect over time on weight (p = 0.19), BMI (p = 0.16), WC (p = 0.84), TSF (p = 0.97), SBP (p = 0.87) and DBP (p = 0.07) in the intervention groups or the control group (Figs. 2, 3, 4, 5, 6 and 7).

## Discussion

The results of our study indicate that soy consumption improves the metabolic status of elderly women. These improvements were more visible in the soy-nut group. However, clinical indices did not show any significant difference between intervention groups and control group. There are many studies on the health effects of soy [12, 19, 20, 34, 39], but only few studies described the use of two types of soy at the same time, on several components of MetS and they often have different outcome or methods [10, 14, 17].

Our study shows that receiving both types of soy for 12 weeks resulted in a significant decrease in lipid profile compared with the control group; except for HDL-C and TG. The results of this intervention are consistent with the results of several comprehensive reviews on soy consumption and cardiovascular health [13, 14, 39–42]. All these reports concluded that there was a significant effect of roasted soy-nut and soy protein containing isoflavones on LDL-C, TC, apoB100, but not on HDL-C and TG concentrations, when consumed at levels similar to this study. However, among some previous reports, only a few studies observed significant changes in the HDL-C level [43]. Their studies showed that the highest effects of soy protein on TC and LDL-C occurred within the short-term period of intervention, whereas improvement in HDL-C was observed, only in long- term period, i.e. more than 12 weeks.

Lipid reduction mechanisms are attributed to several components in soybean including trypsin inhibitors, phytic acid, saponins, aglyconegenistein, fiber, soy storage protein (namely, the globulins 11S and 7S), fatty acid predominantly unsaturated fat and specific amino acid profile (higher ratio of arginine to lysine) [11, 44]. However, some researchers attributed the beneficial effects of soybean on lipid profile to the isoflavone content, while others to soy protein and other soy components [43, 45]. These components may be altered by the processing method of the soy products. The more effects of soy-nuts than TSP on lipids can be related to higher isoflavone levels (Table 1), as well as polyunsaturated fatty acid (PUFA) and some trace components (Additional file 1: Table S2). Frying and texturization, leads to a significant decrease of isoflavone, but interestingly, Messina [11] showed processing of the soy products appeared to have had little effect on their influence on lipid.

Beneficial effects of soy-nut and TSP on glycemia status were also shown in this study. Health benefits of soy on glycemic control have been reported in the most studies on animal models [7, 8]. Epidemiologic studies also explained similar results [16, 46]; however, results from human intervention studies were not compatible [34, 47]. Improvement in blood glucose, insulin secretion and HOMA-IR has been reported in postmenopausal women with abdominal obesity [48], with insulin resistance [15] and or in healthy postmenopausal women [44], as well as in a group of adult men and women with Type 2 diabetes [49, 50], whereas this was not observed in some studies [26, 47]. One explanation of this lack of benefit could be related to the study design. In many non-effective trials, the participants were healthy. Previous evidence on the beneficial effects of soy on serum lipid has shown that soy consumption leads to decrease level of serum lipid only in hyperlipidemic patients and not healthy participants [13]. It can be concluded that no significant effect on glycemic control markers in healthy subject may have been due to normal levels of these markers at the baseline.

Soybean is rich in soluble fiber, tannins, phytates, and genistein, all of which inversely correlate with carbohydrate digestion and glycemic response. Soybean intake also reduces the risk of developing diabetes in the same way it protects against obesity; because it is high in fiber, low in fat, and has low glycemic index [51]. In the current study, the improvement in FBG, insulin and HOMA-IR after the active treatment in the roasted soy-nut group without a change in weight is independent of the action of soy fiber that enhances the feeling of satiety and thus causes an improvement in insulin resistance secondary to weight loss. Soy-nut has a lower glycemic index than TSP, because of the higher phytate and fiber content [52].

In the present study, the participants were in a chronic inflammatory state (CRP ≥ 3 μg/ml). The findings of this study showed that the consumption of either roasted soy-nut or TSP had no significant effect on the concentration of CRP. This result is consistent with several studies investigating the role of different types of tree nuts, peanut, and soy nut consumption [17], soy protein consumed through soy based foods [53, 54] or extracted isoflavones [26] on CRP in postmenopausal women and middle-age men and women. In contrast, some studies did observe a significant decrease in serum CRP, one of which included 104 obese diabetic patients using glucose lowering drugs who consumed a dietary soy supplement for 12 month [55]. Another one included 41 Type 2 diabetic patients with nephropathy who consumed TSP for 4 years [18] and Liu et al. [56] examined the effect of whole soy (soy flour) and purified daidzein on postmenopausal women who were equol producers for 6 month. The inconsistency between those studies could be due to differences in the study samples, such as equol producers or use of medications, as these studies included subjects who had diabetic complications, compared to the participants in the current study who were free from secondary MetS complications and did not use any glycemic or lipid-lowering medications.

Studies on the effects of soy on fibrinogen are limited. However, the lack of significant effect of soy consumption on serum fibrinogen in the current study is consistent with the majority of previous studies that have examined the effect of soy for time periods ranging from 4 weeks to 6 months in 47 healthy postmenopausal women [20], 69 mildly hypercholesterolemia perimenopausal women [25] and 20 Type 2 diabetic subjects [57] or 20 young healthy normolipidemic subjects [58]. Only one of the previous studies of 389 osteopenic, postmenopausal women has reported the significant decrease of fibrinogen with soy isoflavone supplement intake. They found that daily intake of 54 mg genisteinplus calcium, vitamin D3, and a healthy diet for 24 months resulted in a significant reduction in serum fibrinogen [19]. The reason of the difference may be because of long-term intervention.

The beneficial effects of both types of soy on TAC and MDA as indicators of lipid peroxidation and antioxidant activity were shown in this study. The compounds in soybean with antioxidant properties including flavonoids, isoflavones (genistein and daidzein), tri-terpenoids, carotenoids, tocopherols, and saponins may protect against lipid oxidation and improve total antioxidant status [59]. Linoleic acid is also a predominant fatty acid of soy food and has specific effects on eicosanoid synthesis and metabolism and lipid peroxidation [60].

The health benefits of soy on suppressing LDL oxidation by genistin were shown in vitro and animal studies [9, 10, 12]. Human-based results were controversial. Fritz et al. [61] examined antioxidant effect of three levels of isoflavones among women. Soy leads to increase in antioxidant activity regardless of the level of isoflavone consumed. Swain et al. [62] also showed that the consumption of soy protein could affect TAC plasma after 12 weeks. The study among diabetic retinopathy patients showed reduced levels of lipid oxidation by daily supplementation of 120 mg of genistein and 57 mg of daidzein, for 12 weeks [21]. In contrast, some studies did not observe significant improvement in oxidative stress markers. One of which included 25 type 2 diabetic patients with nephropathy who consumed soy milk supplement for 4 weeks [22] and the other which included 43 oophorectomized women who consumed 75 mg/day isoflavones tablet for 12 weeks [52]. The inconsistency between studies could be due to the form in which soy was provided. The isoflavones in soy milk are mainly hydrophilic ß-glycoside conjugates and therefore, faster absorption rates and earlier peak serum concentrations are expected for all subjects given soy milk compared with the solid soy food matrix of TSP [63].

Researchers believed that antioxidant activity of soy may be due to its phytoestrogen or high phytic acid content [59]. The phytate in soy may quench free radicals because of its metal chelating ability [64]. Nevertheless, Engelman et al. [65] showed that neither isoflavones nor phytate in soy protein isolate had a significant effect in reducing oxidative damage. According to the results, despite the higher phytoestrogen content of roasted soy-nut, both soy types caused a significant improvement on MDA and TAC indicators. It seems that processing of the soy products appeared to have little effect on their influence on oxidative stress markers.

The lack of a significant effect in clinical indices including WC, TSP, SBP and DBP in the current study is consistent with the majority of studies carried out among postmenopausal women that have examined the effect of soy for time periods ranging from 12 weeks to 12 months and observed no significant reduction in anthropometric measurements [24, 27, 66]. In contrast, some others reported significant reduction on weight, BMI and WC [67–69]. The reason for this difference could be due to the simultaneous use of lifestyle improvement programs and the presence of subjects in different age and gender groups.

This study may have some limitations. First, we did not include a placebo in the study since the participants in the treatment groups were consuming natural soy products, not soy supplement pills. It was impossible to apply any placebo for the control group. Second, we attempted to follow the participants and used a dietary and physical activity record. However, dietary assessment might have some biases. Third, we could not measure serum or urine isoflavones levels, which could be a limitation of this study. The inclusion of only subjects with no need for medication is a strength point of this study, because it allowed for focusing on the potential of soy to act as a prevention strategy, to possibly prevent or delay the need for pharmaceutical treatment. Our participants had a kind of homogeneity in sex, age and health conditions, too. Also we have a high compliance of participants who completed this study. Considering, the use of two types of soy (natural and processed form), and the effects of both kinds in improving MetS’ features, can help us in providing different types of soy products according to people’s taste.

## Conclusion

This study showed the beneficial effect of soy in elderly women with borderline parameters of MetS who suffered from a hyperlipidemic, insulin resistance status (TG/HDL-C > 3.5, HOMA-IR > 2.5 and fasting insulin > 12.5) and oxidative stress (MDA ≥ 5). Soy-nut decreases these indicators in a greater degree than TSP. The more effects of soy-nut can be due to the higher presence of some components, such as isoflavone genistein, PUFA, and some trace components, and also lower glycemic index than TSP. Therefore, inclusion of soy in their usual diet can reduce the need for medical treatment through the simultaneous improvement of multiple metabolic disorders. Another important finding is that although the manufacturing process may affect the beneficial effects of soy products, processed products, as TSP, can also provide beneficial effects for consumers.

## Additional file

**Additional file 1: Table S1.** Estimated energy, macronutrient and fiber intakes at baseline and after the intervention in elderly women with MetS. **Table S2.** Mean of dietary intake of the participants during the 12-week intervention by Generalized Linear Model repeated measures. **Table S3.** Estimated physical activity levels at baseline and after intervention in elderly women with MetS.

## Data Availability

We have had access to all the data in the study (for original research articles) and accept responsibility for its validity.

## Abbreviations

MetS: metabolic syndrome
TSP: textured soy protein
TC: cholesterol
TG: triglyceride
HDL-C: high density lipoprotein
LDL-C: low density lipoprotein
VLDL-C: very low density lipoprotein
Apo B100: apolipoprotein B100
APO AI: apolipoprotein AI
MDA: malondialdehyde
TAC: total antioxidant capacity
TBARS: thiobarbituric acid reactive substances
FBG: fasting blood glocuse
HOMA-IR: homeostasis model of assessment insulin resistance
CRP: C-reactive protein
WC: waist circumference
TSF: triceps skin fold
SBP: systolic blood pressure
DBP: diastolic blood pressure
GLM: Generalized Linear Model
CVD: cardiovascular disease
ROS: reactive oxygen species
IPAQ: International Physical Activity Questionnaires
BUMS: Babol University of Medical Sciences
