# Supplementary material for: Clinical and metabolic response to soy administration in older women with metabolic syndrome: a randomized controlled trial
Source: Diabetol Metab Syndr. 2019 Jun 20;11:47. doi: 10.1186/s13098-019-0441-y (PMC6584999; doi:10.1186/s13098-019-0441-y)
Supplement: Supplementary file 1 — Additional file 1: Table S1. Estimated energy, macronutrient and fiber intakes at baseline and after the intervention in elderly women with MetS. Table S2. Mean of dietary intake of the participants during the 12-week intervention by Generalized Linear Model repeated measures. Table S3. Estimated physical activity levels at baseline and after intervention in elderly women with MetS. [file 13098_2019_441_MOESM1_ESM.docx]

**Table S1.** Estimated Energy, Macronutrient and Fiber Intakes at Baseline and after the Intervention in Elderly Women with MetS

| **Nutrients** | **Baseline** | | | **p-**  **value** | **After Intervention** | | | **p-value** | **Mean Change^1^** | | | **p-value** |
| --- | --- | --- | --- | --- | --- | --- | --- | --- | --- | --- | --- | --- |
|  | **Soy-nut** | **TSP** | **Control** |  | **Soy-nut** | **TSP** | **Control** |  | **Soy-nut** | **TSP** | **Control** |  |
| **Total Energy^1^ (kcal)** | 1950±8 | 1941±7 | 1956±7 | 0.35 | 1957±7 | 1959±7 | 1970±5 | **0.23** | 6.3±1.28 | 17.5±2.76 | 13.5±2.38 | 0.41 |
| CHO (g) | 275.4±1.56 | 276.7±1.74 | 278.7±1.27 | 0.34 | 278.0±1.47 | 278.7±1.50 | 281.2±1.19 | 0.33 | 2.5±0.35 | 2.0±1.2 | 2.4±1.32 | 0.93 |
| Protein (g) | 75.1±0.59 | 73.7±0.50 | 73.8±0.70 | 0.21 | 73.5±0.68 | 75.2±0.50 | 74.0±0.70 | 0.35 | -1.6±0.38 | 1.4±020 | 0.24±0.04 | 0.28 |
| Total Fat (g) | 60.9±0.23 | 59.9±0.25 | 60.6±0.28 | 0.18 | 61.2±0.25 | 60.4±0.25 | 61.0±0.21 | 0.19 | 0.35±0.21 | 0.56±0.20 | 0.40±0.20 | 0.17 |
| SFA (g) | 16.4±0.10 | 16.1±0.09 | 16.3±0.09 | 0.11 | 16.3±0.11 | 16.5±0.10 | 16.2±0.13 | 0.22 | -0.12±0.09 | 0.34 ±0.11 | -0.09±0.12 | 0.30 |
| MUFA (g) | 26.5±0.12 | 26.0±0.10 | 26.2±0.13 | 0.32 | 26.7±0.16 | 25.8±0.10 | 26.4.±0.13 | 0.38 | 0.20±0.08 | -0.26±0.09 | 0.19±0.08 | 0.40 |
| PUFA (g) | 18.0±0.12 | 17.8±0.13 | 18.1±0.11 | 0.30 | 18.2±0.72 | 18.1±0.11 | 18.4±0.10 | 0.24 | 0.20±0.10 | 0.30±0.11 | 0.33 ±0.10 | 0.69 |
| Fiber^2^ (g) | 25.9±0.14 | 26.1±0.13 | 26.2±0.15 | 0.22 | 26.2±0.14 | 25.6±0.16 | 26.7±0.17 | 0.46 | 0.29±0.09 | -0.47±0.10 | 0.48±0.10 | 0.58 |

Note. TSP, textured soy protein; CHO, carbohydrate; SFA, saturated fatty acid; MUFA, monounsaturated fatty acid; PUFA, polyunsaturated fatty acid.

Values are mean± standard error, n = 75 (each group of 25 participants).

1 Energy from the soy supplementations was taken out.

2 Fiber from the soy supplementations was taken out.

The values are an estimate of daily intakes that are self-reported.

P-values show difference between groups (ANOVA), (p<0.05).

**Table S2.** Mean of dietary intake of the participants during the 12-week intervention by Generalized Linear Model repeated measures

| ***Nutrients*** | ***Treatment Group*** | | ***Control***  ***(n=25)*** | **P.value*** |
| --- | --- | --- | --- | --- |
|  | ***Soy-nut***  ***(n=25)*** | ***TSP***  ***(n=25)*** |  |  |
| **Total Energy (kcal)** | 1943.0± 50.45 | 1939.0±53.39 | 1959.0±54.65 | 0.52 |
| **CHO (g)** | 267.2±30.12 | 275.3±31.14 | 277.2±30.11 | *0.35* |
| **(% of energy)** | 55.0± 3.19 | 56.8±4.21 | 56.6±3.18 |  |
| **Protein (g)** | 74.9±8.24 | 81.4±9.24 | 79.8±8.52 | *0.24* |
| **(% of energy)** | 15.4±3.01 | 16.8±3.34 | 16.3±3.15 |  |
| **Total Fat (g)** | 63. 9±9.11^a^ | 56.87±8.52 | 59±9.73 | <0.05 |
| **(% of energy)** | 29.6± 5.65 | 26.4±5.35 | 27.1±5.95 |  |
| **SFA (g)** | 11.9±3.81^a^ | 11.6±2.92^a^ | 16.5±4.73 | <0.001 |
| **(% of energy)** | 5.5 ±1.78 | 5.4±1.31 | 7.6±2.21 |  |
| **MUFA (g)** | 20.0±5.21 | 20.5±5.45 | 19.6±5.18 | 0.51 |
| **(% of energy)** | 9.25±1.56 | 9.5 ±1.68 | 9.0±1.54 |  |
| **PUFA (g)** | 31.9±7.42^ab^ | 24.8±6.58 | 22.8±6.19 | <0.001 |
| **(% of energy)** | 14.8±2.86 | 11.5±2.44 | 10.5±2.24 |  |
| **Fiber (g)** | 40.8±8.24^a^ | 39.3±9.21^a^ | 25.3±5.13 | <0.001 |
| **Phosphorus (mg)** | 803.0±20.41^ab^ | 510.0±21.11 | 400.2±19.04 | <0.001 |
| **Potassium (mg)** | 3684.1±176.44^ab^ | 2420.2±179.56 | 2297.0±185.32 | <0.001 |
| **Calcium (mg)** | 1110.2±92.34^a^ | 1080.2±88.45^a^ | 770.1±76.41 | <0.001 |
| **Folic Acid (mcg)** | 170.1±30.15 | 168.8±27.11 | 170.8±29.14 | *0.82* |
| **Magnesium (mg)** | 450.3±5.22^ab^ | 320.1±5.31 | 300.0±5.06 | <0.001 |
| **Zinc (mg)** | 11.8±5.62 | 10.2±3.91 | 10.9±4.73 | *0.14* |
| **Iron (mg)** | 20.7±6.61 | 19.7±6.91 | 20.3±7.8 | *0.27* |
| **Vit A(RE)** | 8282.1±65.12 | 8380.3±60.21 | 8250.5±66.35 | *0.38* |
| **Vit E (mg)** | 8.4±1.51 | 8.2±1.52 | 8.6±1.41 | *0.22* |
| **Vit C (mg)** | 70.9±12.14 | 69.1±12.15 | 72.1±12.11 | *0.87* |
| **Vit B1 (mg)** | 1.1±0.49 | 1.2±0.67 | 1.0±0.59 | *0.52* |
| **Vit B2 (mg)** | 1.7±0.47 | 1.4±0.51 | 1.9±0.42 | *0.29* |
| **Vit B6 (mg)** | 0.76±0.11 | 0.71±0.09 | 0.92±0.12 | *0.27* |
| **VitB12 (mg)** | 2.1±0.62 | 1.9±0.71 | *2.1*±0.60 | *0.26* |

Note. TSP, textured soy protein; SE, standard Error; CHO, carbohydrate; SFA, saturated fatty acid; MUFA, monounsaturatedfatty acid; PUFA, polyunsaturated fatty acid.

Food intake was analysed according to the Iranian food composition table.

*Significant time x groups interaction effect (P<0.05) (The Generalized Linear Model (GLM) repeated measures analysis, two factor mixed design was applied to detect the changes in mean of dietary intake of the participants during the 12-week intervention).

a. Significantly different *w*ith the control group

b.Significantly different *w*ith the TSP group

**Table S3.** Estimated Physical Activity Levels at Baseline and after Intervention in Elderly Women with MetS

| **Physical**  **Activity Domains^a^** | **Baseline** | | | | | **After Intervention** | | | | | | **Mean Change^1^** | | | | | | | |
| --- | --- | --- | --- | --- | --- | --- | --- | --- | --- | --- | --- | --- | --- | --- | --- | --- | --- | --- | --- |
|  | **Soy-nut** | **TSP** | **Control** | **p-value** | | **Soy-nut** | | **TSP** | | **Control** | **p-value** | **Soy-nut** | | **TSP** | | **Control** | | **p-value** | |
| Activities  at work | 662.9±7.31 | 645.0±7.42 | 665.0±9.01 | 0.15 | 670.2±6.80 | | 655.2±8.2 | | 660.8±9.95 | | 0.27 | 7.2±2.36 | 10.2±2.70 | | -4.2±1.94 | | 0.51 | |  |
| Activities for transportation | 452.5±14.29 | 455.4±14.70 | 441.6±15.2 | 0.82 | 458.6±14.09 | | 448.7±16.06 | | 439.0±13.55 | | 0.64 | 6.06±1.85 | -6.7±1.06 | | -2.5±0.99 | | 0.79 | |  |
| Activities for domestic and garden | 1602.5±96.26 | 1585.0±94.16 | 1627.8±59.33 | 0.94 | 1596.8±98.29 | | 1592.7±80.08 | | 1630.5±70.54 | | 0.92 | -5.7±1.63 | 7.6±2.21 | | 2.7±0.53 | | 0.93 | |  |
| Leisure time activities | - | 10.4±0.99 | - | - | - | | 10.8±1.45 | | - | |  | - | 0.4±0.03 | | - | | - | |  |
| Total physical activity score | 2717.9±99.07 | 2695.9±98.71 | 2734.5±70.68 | 0.93 | 2725.7±100.8 | | 2707.4±83.94 | | 2730.3±66.98 | | 0.91 | 7.6±1.44 | 11.5±3.42 | | -4.2±0.50 | | 0.98 | |  |

Note. TSP, textured soy protein.

Values are mean± standard error, n = 75 (each group of 25 participants).

^a^ Expressed as MET-minutes per week: Met level × minutes of activity/day × days per week.

^1^ For difference in mean changes between the groups.

The values are an estimate of weekly physical activity that is self-reported.

P-values show difference between groups (ANOVA), (p<0.05).
